# Dr. C. J. B. Williams on Constipation

**Published:** 1845-01

**Authors:** 


*Dr. C. J. B. Williams on Constipation.*—We now pass on to the consideration of that form of *constipation* which corresponds with indigestion. This affection is principally seated in the colon, and may be regarded as opposed to diarrhœa; it may consist in defective secretion of the canal, or defective muscular action, or both combined; and each of these, defective secretion and defective muscular action, may depend on different pathological causes. Generally speaking, defective secretion and defective muscular action go together, just as excessive secretion and excessive muscular action of the canal constitute diarrhœa. They produce torpor of the bowels, and extreme irregularity in their action.

Now, among the *causes* to which we may trace this imperfect action, we find, in the first place, the character of the food to be the main source of costiveness. For example, food of a certain nature will produce fæculent matter, but does not, by its presence, excite the biliary secretion, or the peristaltic action of the bowels. This is the character of all farinaceous food generally; more particularly of those kinds which abound in starch, as arrow-root, gluten, and rice. Rice and arrow-root we find are constipating, not from any astringent property they possess, but merely negatively so, from the want of any stimulating property. Fæculent matter, formed in large quantity, tends to lessen, rather than increase the peristaltic action; and the bowels are liable to become costive from the mechanical obstruction offered to the passage of the food. Further, the food may be astringent, by having the effect of arresting the secretions of the canal, or else by its acrid and irritating quality, causing spasmodic contractions, as is sometimes the case in colic. Some liquids, such as vinegar in any considerable quantity, very often produce costiveness of the bowels, at the same time that they greatly irritate the passages.

Secondly, we may trace constipation to torpor or imperfect action of the intestinal muscles; and, perhaps, not only of the muscles, but also of the secretory power of the intestines; for the intestinal canal is not only a moving organ, but also a secretory organ. This is illustrated after the operation of a strong purgative; the bowels generally become torpid, and inasmuch as the intestines become exhausted, and the secretions otherwise have been excited too freely, they seem to secrete less freely afterwards. But, sometimes, it is a sign of general weakness of the intestinal muscles, a part of the general weakness of the system; and, accordingly, in anæmia, you find torpor of the bowels taking place. The same thing is observed in any state of great weakness, after fevers and illnesses, and after extreme fatigue of the body. Paralysis, and particularly paraplegia, are causes of this. Some other causes act in a similar way. Enervating heat, exhausting the body, greatly reduces the powers of the intestines. Continued cold has a similar effect. Moderate cold, however, increases the tonic action of the intestines to a high degree. In other conditions—the state of atonic plethora, for example—a costive state of the bowels is frequently present. The excretions are often defective from irritability of the muscles of the intestines.

Thirdly, this morbid condition may arise more directly from deficient secretion, rather than defective peristaltic action, which latter may go on, even if the secretion be imperfect; a scanty evacuation is the result. This is to be met with in connection with defective secretion of the liver, more particularly; arising from different diseases of that organ interrupting its secretion, or from obstruction of the ducts passing out of it. Diseased states, likewise, of the intestine, such as congestion of its coats, connected with derangement of the liver, are often attended with this sort of defective secretion. Inflammation may be considered as, in some degree, interfering with the powers of secretion of the liver and the intestines. Enteritis is one of the instances in which the secretion appears to be greatly interrupted. There is, here, not merely spasmodic contraction, but the secretion is interfered with. So, with regard to gastritis, where there is much irritation, it frequently happens that the lower part of the canal is defective in excitability and in secretion. Defective secretion and imperfect contractility are frequently combined together.

Now, from this you may understand that constipation may be either symptomatic of various diseases, or it may be idiopathic—itself the chief cause of disease. The frequency of the occurrence of symptomatic constipation is one great reason why purgatives are so often useful in the treatment of diseases of almost every kind; for, in a majority of cases, there is symptomatic costiveness, and a tendency to great constipation of the bowels. It thus becomes an indication to use purgatives; not merely to remove this state, but, likewise, because the accumulation of the fæces becomes a source of still further mischief, aggravating greatly the organic disease, and adding to the local and general symptoms. In febrile diseases, there is apt to be a reaction, and it often happens that the evacuation of the loaded bowels very much mitigates this effect. Idiopathic constipation, where it exists without any other permanent disorder, is not uncommon, and, in a great number of cases, it, perhaps, consists in an insufficient action of the lower intestines, or of the colon. Sometimes, in costiveness, there is distention of the bowels, especially of the colon, by flatus, causing a feeling of fulness and tightness in the abdomen. There is often, too, considerable tympanitic distention, where wind is secreted in large quantities. Accordingly, the sounds arising from the motion of wind in the intestines are commonly heard in persons of a constipated habit. There are shifting or aching pains felt in various parts of the abdomen, usually, perhaps, in the right iliac, or hypochondriac region, about the right and sometimes the left hip, and in the loins; also, uneasy feelings, such as restlessness, and inability to be quiet, without being able to explain exactly why. In children, symptoms of chorea sometimes arise from a costive state of the bowels; and, in adults, there are cramps of the legs, which are very common signs connected with imperfect action of the bowels. Other symptoms arise in the general system; headache is a very common sign, and there is, in general, languor and depression of spirits. These different symptoms arise partly from the direct irritation of the accumulated fæces on the nerves, and partly from the noxious influence of the retained and absorbed excrement, for matters long retained in the alimentary canal, are partly absorbed again into the system. The fæces are in a compact and dry state, if the bowels are not regularly evacuated, which shows that the moister parts are absorbed away; and those persons who have habitual costiveness of the bowels, generally have unpleasant smells about them, and the countenance is more sallow than usual; the tongue is usually more or less loaded, and the breath is very fœtid; the fæces, too, are darker than usual, and they present a moulded form from the cells of the colon, or the different portions of the intestines in which they have been retained. The accumulation of fæces in the abdomen may cause a considerable amount of swelling, and various other symptoms; for example, by their pressure, as not unfrequently happens, on the gall-ducts, they may produce jaundice; or, on the stomach, nausea, and a disturbance of the function of digestion, or dyspepsia; and, frequently, hæmorrhoidal swellings, or piles, are caused by their presence. In extreme cases, numbness and swelling of the legs, and even paralysis, have been induced by their pressure on the nerves of the pelvis. Sometimes the abdomen is enormously distended; and I have seen such patients, who have had the appearance of advanced degrees of dropsy, and who have been pronounced to be laboring under organic disease, and tumors of various kinds. I remember the case of a patient who, when brought into the hospital, was supposed to be affected with organic disease of the abdomen, and had been treated for such for some time, in whom the abdomen had been distended for six or eight weeks, to a very great degree, insomuch that the cartilages of the lower ribs were turned out, and yet all this tumor vanished, after a purgation of some days, and was produced solely by a vast accumulation of fæculent matter. Neglected constipation will lead to local irritation, and, as a result, you may have diarrhœa set up, from the irritation of the parts, and increased secretion results. Dysentery even may ensue, and inflammation of the colon, attended with pains of various kinds; neuralgia and severe griping pains of the bowels, accompanied with colic, and a spasmodic contraction, ending in deep-seated enteritis, and cholera. These are the local effects. Organic changes, too, may arise; and, in addition, a cachectic state of the whole system, a low febrile state, from the bad influence of the accumulated fæces on the nervous system.

The *predisposing causes* you may deduce from what I have already said, as to the pathological characters of constipation. Sedentary habits, especially when combined with various enervating influences, greatly promote constipation. This occurs very often among females who are much confined within doors, and who are in the habit of using but little exercise. It is sometimes excited by attacks of hysteria, and, as I said before, by bad or defective diet. Particular kinds of waters, those abounding in iron and calcareous matters greatly aid in producing constipation. All the different causes that produce general weakness may cause costiveness. Excessive action of purgatives, and neglect of the bowels, will often produce it. But, perhaps, one of the most frequent causes, which it is important to remember, is inattention to the calls of nature, or want of regularity in the evacuation of the bowels. It is a process that ought always to be considered as conducive to health, and should be set apart to be done at a regular time in the day, quite as much so as the eating of any given meal.

Now, with regard to *the treatment.* The first indication of the common form of constipation, where there is an accumulation of fæces, is to unload the bowels; and to fulfil this, purgatives and injections are the chief means. The best purgatives for this purpose are the mixed purgatives, those which exercise an action on the whole canal; for, although costiveness often proceeds from the defect of one part of the canal, the torpor ultimately extends to the whole; and it is not a safe practice to administer too strong a purgative of one kind, to act on one part of the canal only. The effect of giving a large dose of aloes, or a large dose of colocynth alone, is to irritate one part of the canal, and leave the liver unaffected, and thus, perhaps, convert constipation into inflammation. For this reason it is highly advantageous to combine the different purgatives together; and if there is already much pain and irritation produced by the constipation, we should combine with purgatives those medicines which deaden the sensibility, and prevent spasm. For this reason, the combination of calomel, colocynth, rhubarb, and henbane, answers well. If these are not sufficient, great advantage will result from the addition of occasional doses of castor oil, or else of salts and senna, in the form of a black draught. I cannot dwell too strongly on the virtues of castor oil in cases of prolonged constipation. If given in large doses, it does not irritate as other medicines; yet it acts more thoroughly; it penetrates, and, in some degrees, has the mechanical power of insinuating itself into the crevices and curvatures of the intestines, and thus dislodging the accumulated masses, where calomel and other medicines had failed to act. Mercury, though it acts more especially on the liver, and stimulates the secretion of bile, is accompanied with a great deal of griping pain, and is not, by itself, so effective in unloading the bowels. Where the accumulation is very considerable, it is desirable to facilitate the action of the bowels by copious injections of oil or soap and water. Remember that these accumulations of fæculent matter sometimes consist of a hard and compact mass—so hard that purgatives, which merely stimulate the canal to contraction, produce very little effect upon it. Injections of castor oil, with a little turpentine, or of olive and linseed oil, mixed up with gruel, are useful; of this one, two, three, or four pints may be thrown up. The use of turpentine is indicated where there is a tympanitic distention of the abdomen. Rue and asafœtida injections are also useful in such cases. After the fæces have been dissolved, the bowels pass into an irritable state, and there is often a considerable amount of pain. It frequently happens that, after the great accumulation has been removed, the tongue is more furred, the pulse more excited, and the system more deranged. In this state of things, mercurial purgatives, combined with opium, or mercury and Dover's powder, followed by small doses of castor oil, or else rhubarb, or some other mild aperient, may be given, for several days afterwards, with great advantage. If the opium irritates, instead of giving Dover's powder, conium may be employed as a substitute; moderate doses of conium and mild aperients may be given until the bowels are brought into regular action. Where there is a tendency to low inflammatory affections, such as gastritic dyspepsia, which often occurs in costive habits, the aperient that suits best will be found to be small doses of castor oil; a teaspoonful, the first thing in the morning, or the last thing at night, acts admirably, and ensures the regular evacuation of the bowels. In some persons olive oil acts in the same way. Castor oil is extremely valuable in cases of dyspepsia, where stronger purgatives do mischief. Sulphate of potash and sulphur sometimes answer very well.

The diet of persons disposed to constipation should be of a plain character, not too much abounding in farinaceous food. Too much bread should be avoided. There is a common error with respect to bread, and many persons will eat it all day long; hence, it becomes a great cause of constipation. It is better to eat it two or three times a day, in small quantities. A thing you will find of great use—and which is very popular in the sister-kingdom—is oatmeal porridge. It has none of the constipating effects of farinaceous food in general. Fruit is very often taken with a view to counteract costiveness, but I believe its effect has been overrated. The most important part of the treatment of constipation consists in preventing the accumulation of fæculent matter; and, therefore, the promoting of the proper and natural action of the bowels is far better than any description of diet or medicine that can be given. I must say a few words upon the practice fallen into of late, by a good many individuals, namely: that of confining themselves to the use of injections to promote the action of the bowels. I think this practice is a very injurious one; for, when injections are frequently administered, instead of producing a healthy action in the bowels, they generally tend to bring it into a state of torpor. They mechanically distend the bowels, and thus do mischief. General tonic measures, to improve the health, are sometimes necessary. The application of electricity, or galvanism, has been recommended by some to promote the action of the bowels; it may be useful so far as excitement of the peristaltic action is concerned.

*Boston Med. and Surg. Jour.*, from *Lon. Med. Times.*

